# Tunneling-induced optical limiting in quantum dot molecules

**DOI:** 10.1038/s41598-020-73343-2

**Published:** 2020-10-01

**Authors:** Mohadeseh Veisi, Seyedeh Hamideh Kazemi, Mohammad Mahmoudi

**Affiliations:** Department of Physics, University of Zanjan, University Blvd., 45371-38791 Zanjan, Iran

**Keywords:** Optics and photonics, Optical physics, Nonlinear optics, Nanoscience and technology, Nanoscale materials, Quantum dots

## Abstract

We present a convenient way to obtain an optical power limiting behavior in a quantum dot molecule system, induced by an interdot tunneling. Also, the effect of system parameters on the limiting performance is investigated; interestingly, the tunneling rate can affect the limiting performance of the system so that the threshold of the limiting behavior can be a function of the input voltage, allowing the optimization of the limiting action. Furthermore, by investigating the absorption of the probe field, it is demonstrated that the optical limiting is due to a reverse saturable absorption mechanism; indeed, analytical results show that this mechanism is based on a cross-Kerr optical nonlinearity induced by the tunneling. Additionally, the limiting properties of the system are studied by using a Z-scan technique.

## Introduction

Rapid development in laser technology has made it possible to generate extremely short and intense laser pulses, which pose a potential hazard for optical instruments and human eyes. Simultaneously, studies on optical power limiting become more important, due to its applications for protection of sensitive optical instruments from exposure to intense laser beams, and the quest for optical limiters (OLs) has been a topic of great scientific and practical interest^1–6^. Optical power limiting occurs when the absolute transmittance of a material decreases as the intensity of the input laser field increases beyond a critical value; indeed, an optical power limiter exhibits a high and linear transmission (at least of 70$\%$) below a certain threshold, to preserve the detection function of sensors, but above which the optical nonlinear properties of the material limit the transmission of light. Desirable properties for power limiting applications can include a high linear transmittance, a broad spectral response, and a low limiting threshold^4^. Noting that the limiting threshold is defined as the incident intensity at which the transmittance falls to 50$\%$ of the linear transmittance.

Due to the widespread use of intense lasers in various fields such as the scientific, industrial, medical therapy and military sectors, the power limiting becomes an important research branch in nonlinear optical processes; materials possessing large optical nonlinearities have been potentially employed in a number of applications in limiting devices and extensive theoretical and experimental investigations on power limiting, based on optical nonlinear processes, have been reported^7–12^. One of the mechanisms that may lead to a nonlinear absorption effect is reverse saturable absorption (RSA), also referred to as excited-state or two-photon absorption, in which the absorption cross section from excited-state energy levels is significantly higher than the ground-state absorption cross section^4,8,13^; in fact, RSA, being one of the major OL mechanism, has been reported for applications in efficient OLs^14–20^, with compounds such as organic and inorganic materials^21,22^, liquid crystal^23^, nanocomposites^24^, nanoparticles^25^ and thin film^26^.

Quantum dots (QDs)-semiconductor nanostructures that confine the motion of conduction band electrons in all three spatial directions so that electrons and holes can occupy only the set of discrete energies-have attracted tremendous attention from scientists over the past four decades; because of the size-tunable atomic-like properties, along with their high nonlinear optical coefficients and great flexibilities in device design by choosing materials and structure dimensions, QDs have been intensively investigated for their unique structural and optical behaviors^27,28^. It is well-known that the transitions between different electronic states in QD nanostructures can be optically excited by laser fields. In this context, studying nonlinear optical process, including RSA and saturable absorption (SA) have been pursued by several researchers^29–32^; for example, the intensity-dependent nonlinear absorption and nonlinear refraction of QDs were experimentally investigated in Ref.^29^. When two or more closely-spaced individual QDs- with significant interdot coupling- are combined, a quantum dot molecule (QDM) is formed by the tunneling interaction among QDs, in which an electron can pass through the potential barrier between quantum dots via the interdot tunneling^33^. Such molecules are applicable volunteers for quantum coherence-based studies, because of ease of integration, controlling size and energy level spacing as well as their large bandwidth^34^. In this regard, it has been shown that the optical properties of such systems can depend on the interdot tunneling effect, which has been used for controlling optical bistability^35^, entanglement^36,37^, light propagation^38,39^ and orbital angular momentum transfer^40^. Notice that the QDMs cannot exhibit vibrational or rotational features and they indeed display somewhat different properties with respect to usual molecules.

On the other hand, the Z-scan technique is a sensitive and simple method for the determination of intensity-dependent optical properties of materials, and therefore, can determine the limiting properties of systems; in fact, this technique, which was first introduced by Sheik-Bahae et al.^41^, has been used for characterizing the nonlinear optical properties of materials (e.g., nonlinear absorption, refraction or scattering)^42,43^. The Z-scan technique simply involves the measurement of the sign and magnitude of real and imaginary parts of nonlinear susceptibility, via investigating the intensity-dependent variation of optical properties of the sample. Briefly, in a typical Z-scan experimental setup, a laser beam with a transverse Gaussian profile is focused using a lens. The sample is then moving along the propagation direction of the beam, and the transmittance (transmitted energy divided by the input energy) of the sample is determined during its translation; at the focal point, the intensity of the laser beam increases substantially and the sample experiences maximum pump intensity, which gradually decreases in both directions from the focus. Interaction between such a laser beam and the nonlinear material may result in the increase or decrease of the transmittance. Noting that the nonlinear absorption is classified into two types: SA and RSA; in the SA mechanism, the nonlinear absorption decreases by increasing the intensity, leading to a peak in transmission near the focus. Whereas, the nonlinear absorption in the latter increases and therefore a dip in the vicinity of the focal point occurs.

In this paper, we present a convenient way to generate an optical power limiting in a four-level QDM system being induced by the interdot tunneling which can be simply controlled by applying a gate voltage. Also, by solving density-matrix and field-propagation equations, we theoretically investigate the effect of various parameters, including the detunings and the tunneling rate on the limiting effect; by choosing appropriate parameters, the system reveals good OL properties in a certain intensity region which can be attributed to a RSA mechanism. Also, it has been shown how the interdot tunneling can affect the optical limiting performance of the system and the threshold of the limiting behavior can be lowered by increasing the tunneling coupling; indeed, threshold would be a function of the input voltage, allowing the optimization of the OL behavior. In addition, this paper deals with further characterization of the optical power limiting, using a Z-scan technique. Such a controllable limiting behavior, associated to the high linear transmittance at low intensities and the low-threshold OL behavior, may make these molecules promising for OL device development.

## Model and equations

Let us consider a couple of lateral self-assembled (In,Ga)As/GaAs quantum dots with different band structures; the self-assembled lateral QDMs can be produced by the combination of molecular beam epitaxy and atomic layer precise in situ etching on GaAs(001) substrates, which can provide a low density about $5\times 10^7$ cm$^{-2}$ homogeneous ensemble of QDMs consisting of two dots aligned along the $[1\bar{1}0]$ direction^44^. The average lateral size of each QD is almost 9 nm and the interdot barrier thickness is assumed to be few nanometers to occur significant interdot electron tunneling^45^. Noting that applying an external electric field along the molecular (coupling) axis via simple Schottky contacts can control the interdot coupling (more experimental details of the fabrication technique can be found in Ref.^46^).

**Figure 1:**
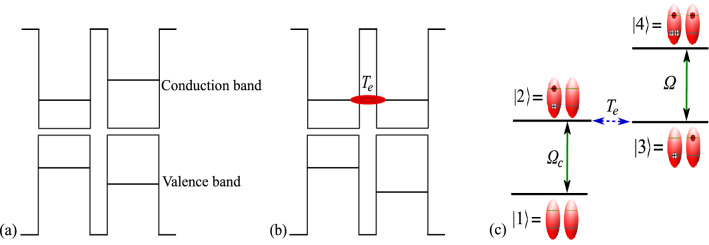
Band structure diagram of the QDM system before (**a**) and after (**b**) applying the external voltage. (**c**) Schematic of the four-level ladder type QDM system and two applied laser fields. The electron and hole are shown by $\ominus$ and $\oplus$, respectively.

In this paper, we consider a four-level cascade configuration for QDM, which is shown in Fig. 1. The ground state defines a level in which two QDs are not excited. The state $|2\rangle$ shows a state that an electron is excited to the conduction band in one of the QDs to generate an exciton in such a way that the other QD does not excited. The electron transfers to the conduction band of the second QD, via the interdot tunneling, to generate an indirect exciton which is shown by the state $|3\rangle$. Finally, the state $|4\rangle$ stands for the state in which the biexciton is established by exciting the electron to the conduction band of the first QD^47,48^. Then, we assume that a coupling laser field of frequency $\omega _{c}$ (with Rabi frequency $\Omega _{c}$) is applied on the transition $\vert 2 \rangle \leftrightarrow \vert 1 \rangle$ while, the transition $\vert 4 \rangle \leftrightarrow \vert 3 \rangle$ is driven by a probe laser field of frequency $\omega _{p}$ (with Rabi frequency $\Omega _{p}$). Worth mentioning is that a similar structure has been proposed for tunneling-induced transparency and related applications^49,50^.

Using rotating-wave and dipole approximations, the Hamiltonian is written as

1 $$\begin{aligned} H_{int}=- \hslash \,\Omega _{c}\,e^{-i\Delta _{2}t}|2>
<1|-\hslash \,\Omega _{p}\,e^{-i\Delta _{4}t}|4>
<3|+\hslash \,T_{e} \, e^{i\omega _{32}t}|3>
<2|+\mathrm {H.c.}, \end{aligned}$$

where the detuning of the fields from the corresponding transitions are given by $\Delta _{2}=\omega _{c}-\omega _{21}$, $\Delta _{4}=\omega _{p}-\omega _{43}$ with $\omega _{ij}$ being as the frequency of the transition $\vert i \rangle \leftrightarrow \vert j \rangle$ (*i* = 2, 4 and *j* = 1, 3). Also, $T_{e}$ is the tunneling matrix element of an electron, representing the magnitude of the coupling between the states $\vert 2 \rangle$ and $\vert 3 \rangle$. Note that the interdot coupling occurs only for two nearly degenerate quantum states, which can be prepared by applying an external static gate voltage to the QDMs. The Rabi frequencies of the coupling and probe fields, respectively, are $\Omega _{c}=E_{c}\mu _{21}/\hslash$ and $\Omega _{p}=E_{p}\mu _{43}/\hslash$ , with $\mu _{ij}$ being as the dipole momentum matrix element from $\vert i \rangle$ to $\vert j \rangle$. Using the density-matrix approach, one can write the following dynamical equations for the system

2 $$\begin{aligned} \dot{\rho }_{11}= & {} i\Omega ^{*}_{c}\rho _{21}-i\Omega _{c}\rho _{12}+\Gamma _{2}\rho _{22},\nonumber \\ \dot{\rho }_{22}= & {} i\Omega _{c}\rho _{12}-i\Omega ^{*}_{c}\rho _{21}-i \,T_{e}\,(e^{-i\omega _{32}t}\rho _{32}-e^{i\omega _{32}t}\rho _{23})-\Gamma _{2}\rho _{22},\nonumber \\ \dot{\rho }_{33}= & {} i\Omega ^{*}_{p}\rho _{43}-i\Omega _{p}\rho _{34}-i \,T_{e}\,(e^{i\omega _{32}t}\rho _{23}-e^{-i\omega _{32}t}\rho _{32})+\Gamma _{4}\rho _{44},\nonumber \\ \dot{\rho }_{21}= & {} i\Omega _{c}(\rho _{11}-\rho _{22})-i\,T_{e}\,e^{-i\omega _{32}t}\rho _{31}-(\frac{\Gamma _{2}}{2}-i\Delta _{2})\,\rho _{21},\nonumber \\ \dot{\rho }_{31}= & {} i\Omega ^{*}_{p}\rho _{41}-i\Omega _{c}\rho _{32}-i\,T_{e}\,e^{i\omega _{32}t}\rho _{21}+i\Delta _{2}\rho _{31},\nonumber \\ \dot{\rho }_{32}= & {} i\Omega ^{*}_{p}\rho _{42}-i\Omega ^{*}_{c}\rho _{31}+i\,T_{e}\,e^{i\omega _{32}t}(\rho _{33}-\rho _{22})-\frac{\Gamma _{2}}{2}\rho _{32},\nonumber \\ \dot{\rho }_{41}= & {} i\Omega _{p}\rho _{31}-i\Omega _{c}\rho _{42}-[\frac{\Gamma _{4}}{2}-i(\Delta _{4}+\Delta _{2})]\,\rho _{41},\nonumber \\ \dot{\rho }_{42}= & {} i\Omega _{p}\rho _{32}-i\Omega ^{*}_{c}\rho _{41}+i\,T_{e}\,e^{i\omega _{32}t}\rho _{43}-[\frac{(\Gamma _{4}+\Gamma _{2})}{2}-i\Delta _{4}]\,\rho _{42},\nonumber \\ \dot{\rho }_{43}= & {} i\Omega _{p}(\rho _{33}-\rho _{44})+i\,T_{e}\,e^{-i\omega _{32}t}\rho _{42}-(\frac{\Gamma _{4}}{2}-i\Delta _{4})\,\rho _{43}. \end{aligned}$$

The remaining equations follow from $\tilde{\rho }_{lm}=\tilde{\rho }_{ml}^* (l,m \in \lbrace 1, \ldots ,4 \rbrace )$ and trace condition. Here, $\Gamma _{i}$ is the decay rate from the level $\vert i \rangle$. It is clear that in general the system does not have a steady-state solution, due to the explicit time dependence of the equations; however, by choosing a zero value for the tunneling detuning ($\omega _{32}$), the coefficients of the above equations do not have an explicit time-dependence exponential factor, and consequently, a stationary steady-state in the long-time limit can be found. Throughout the paper, the tunneling detuning is assumed to be zero or negligible in such a way that the system can have a stationary steady-state.

The polarization vector in the medium is also given by

3 $$\begin{aligned} \vec {P}(z,t)=\chi _{p}\, \vec {\varepsilon }_{p}\,e^{-i(\omega _{p}t-k_{p}z)}+\mathrm {c.c.}, \end{aligned}$$

where $k_{p}=\omega _{p}/c$ and $\vec {\varepsilon }_{p}$ is the incident field amplitude. The susceptibility can be defined as follows $\chi _{p}=\frac{2 \, \vert \mu ^{2}_{43} \vert \, \Gamma _{\mathrm {opt}}}{\hslash \varepsilon _{0} V \,\Omega _{p}}\rho _{43}$, with $\Gamma _{\mathrm {opt}}$ and *V* being as the fraction of the optical power guided in the QDs and the effective mode volume of the single QD, respectively. Noting that the probe transition coherence ($\rho _{43}$) can be calculated from Eq. (2).

In the following, we proceed to investigate the field-propagation equation; under the slowly-varying approximation, the equation for the field propagation can be written as follows

4 $$\begin{aligned} \frac{\partial \varepsilon _{p}}{\partial z}=2\pi i \,k_{p}\,\varepsilon _{p} \,\chi _{p}. \end{aligned}$$

The solution of the above equation is given by $\varepsilon _{p}$(z) = $\varepsilon _{p}(0)\,e^{2\pi i \,k_{p}\chi _{p}z}$. By calculating the steady-state solutions of the rate equations, the transmittance at the propagation distance *z* can be obtained. Finally, the expression for normalized transmittance would be the form of $T=\vert \varepsilon _{p}(l) \vert ^{2}/ \vert \varepsilon _{p}(0) \vert ^{2}$, with $\varepsilon _{p}(0)$ and $\varepsilon _{p}(l)$ being the field at the start of the sample and the field after traversing the sample, respectively. Note that, in the current work, the nonlinear transmittance as a function of input intensity is used to study the power limiting properties.

## Results and discussion

Before presenting the results, it is desirable to point out some important considerations; throughout the paper, we refer to realistic parameters for self-assembled QDs^45^. For simplicity, all the parameters are scaled by the decay rate ($\Gamma _{2}=\Gamma _{4}= \Gamma$) which is about 10 $\mu$eV. The optical confinement factor is considered to be $\Gamma _{\mathrm {opt}}$=2.25 $\times 10^{-3}$^45,48^ and the effective mode volume is *V*=11 nm$^{3}$^51^. As the tunneling rate ($T_e$) depends on the barrier, which determines the decay rate of the electronic states, it is also scaled by the decay rate ($\Gamma$). The wavelength for the transition $\vert 4 \rangle \leftrightarrow \vert 3 \rangle$ is also considered to be 1.36 $\upmu$m. Unless otherwise indicated, a logarithmic scale for input intensities is chosen, in order to show the entire transmittance curve. In the following, we analyze the process of the optical power limiting in a coupled QD system with the tunneling effect, via the investigation of field-propagation and density-matrix equations, i.e., Eqs. (2) and (4); first, the OL properties will be analyzed by plotting the transmittance (transmitted energy/input energy) versus the input intensity. Then, a Z-scan technique will be utilized in order to further characterize the optical power limiting properties.

**Figure 2:**
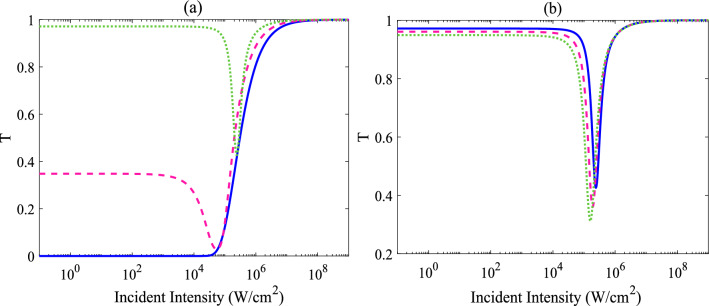
Effects of the detunings on the calculated optical limiting properties. (**a**) Transmittance (T) versus the intensity of the incident field, with $\omega _{23}=0$, $T_{e}=\ 0.2\Gamma$ and different detunings: $\Delta =0$ (solid line), $\Delta =\Gamma$ (dashed line) and $\Delta =2\Gamma$ (dotted line). Plot (**b**) depicts the variation in the normalized transmittance as a function of the input intensity, with $\Delta =2\Gamma$, $\omega _{32}=0$ (solid line), $\omega _{32}=0.3\Gamma$ (dashed line) and $\omega _{32}=0.5\Gamma$ (dotted line).

As the present model contains a number of parameters affecting the optical properties of the system, and subsequently the OL behavior, we first take a glance at the influence of the detunings on the limiting behavior of the system. Figure 2a shows the calculated transmittance versus the incident intensity, with a moderate tunneling rate ($T_{e}= 0.2\Gamma$) and different detunings: Solid, dashed and dotted lines in the figure, respectively, represent the transmitted laser beam intensity (T) for $\Delta _{2}=\Delta _{4}=\Delta =0$, $\Gamma$ and $2\Gamma$. Other used parameters are $\Omega _{c}=0.5 \Gamma$ ($\simeq$ 0.75 $\times 10^{4}$ W/cm$^{2}$) and $\omega _{32}=0$. Notice that the corresponding plot for zero tunneling is omitted, as no apparent variation was observed for its transmission curve. It seems that the one-photon transition (i.e., zero detuning) plays a major role in determining the linear transmittance; by increasing the detunings, as long as it exceeds the one-photon resonance condition, the transmittance at low input intensities increases. Moreover, for the case of zero detunings of both applied fields, in which the one- and two-photon transitions are possible, the molecule does not show the RSA; however, for nonzero and equal detunings, in which only two-photon transition can occur in the QDM, there would be a gradual reduction in transmittance with an increase of the input intensities, indicating an OL effect.

More detailedly, the transmitted laser beam at low input intensities varies linearly with respect to incident input intensities; in fact, the transmittance, and subsequently, the output intensity is proportional to the input intensity, obeying the Beer–Lambert law. But, the transmittance starts to decreases with the increase of the incident intensity so that the transmittance reaches its minimum at a point defined as the limiting amplitude (i.e. the minimum transmission, or equivalently, the maximum output intensity during the region of power limiting effect); it means that the output intensity can be limited by the system. As mentioned previously, power limiting behavior can be found in RSA materials since they become more opaque on exposure to high input intensities. It is worth noting, however, that once RSA action has been started, it does not necessarily continue to all higher intensities^52^; indeed, the absorption of the sample first increases and then decreases by further increasing intensities. Transmittance at higher intensities, here also, is decreased and then starts to increase with stronger irradiance. It is also interesting to note that one can find the improvement in the efficiency of the limiting behavior, by increasing the detuning: In addition to having larger linear transmittance in order to preserve the detection function of sensors, the threshold for the RSA to SA conversion slightly increases by increasing the detuning. As a result, one can expect a better efficiency of the limiting behavior for the case of larger detunings, for which one-photon transitions are completely stopped; so, we continue to use such parameters in the following discussion ($\Delta _2=\Delta _4=2\Gamma$).

Figure 2b depicts the effect of the tunneling detuning ($\omega _{32}$) on the limiting behavior; solid curve shows the transmittance as a function of the incident intensity for a zero tunneling detuning ($\omega _{32}$ = 0), dashed and dotted lines demonstrate the corresponding plot for non-zero tunneling detunings ($\omega _{32}$ = 0.3$\Gamma$ and $\omega _{32}$ = 0.5$\Gamma$, respectively). As the tunneling detuning is considered to be non-zero, a similar limiting behavior is seen; at low input intensities, the transmitted laser beam varies linearly with respect to incident input intensities and then starts to decrease, by further increasing the intensities; after that RSA to SA conversion does occur. As mentioned previously, various parameters used to evaluate the efficiency of OL materials, such as limiting amplitude and limiting threshold (the input intensity at which the transmittance falls to 50% of the linear transmittance); indeed, limiting amplitude is another criteria to describe the limiting performance of materials; a lower value of such a parameter gives a better OL behavior at high fluence. As is clear, at the about same level of linear transmittance, the system possesses prominent optical limiting behavior for all cases. However, choosing different tunneling detuning also produces slightly different limiting amplitude and threshold values for the OL action; a lower limiting threshold and lower limiting amplitude can be found for the case of $\omega _{32}$ = 0.5$\Gamma$ (Notice that linear transmittance for the cases are almost the same: 97%, 96% and 95% for $\omega _{32}=0$, $\omega _{32}=0.3\Gamma$ and $\omega _{32}=0.5\Gamma$, respectively). Here, it is worth reiterating that the nonlinear optical phenomenon, namely, the multi-photon transition plays a crucial role in establishing the OL in the four-level cascade configuration of the QDM. Note that the such a transition can not be possible in the absence of the interdot tunneling.

**Figure 3:**
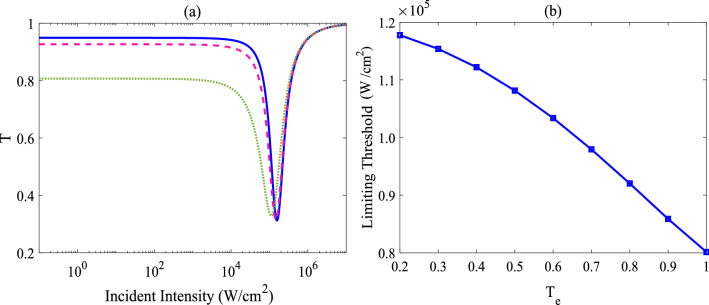
Tunneling affects the optical limiting performance of the system. (**a**) Transmittance versus the intensity of the incident field, for the case of $\omega _{32}$ = 0.5$\Gamma$ and for three different tunneling rates: $T_{e}$ = 0.2$\Gamma$ (solid line), 0.5$\Gamma$ (dashed line) and $\Gamma$ (dotted line). (**b**) Limiting threshold versus the tunneling rate ($T_{e}$). The other parameters are the same as Fig. 2b.

Figure 3a shows the variation in the normalized transmittance as a function of the input intensity, for different tunneling rates: $T_{e}$ = 0.2$\Gamma$ (solid line), 0.5$\Gamma$ (dashed line) and $\Gamma$ (dotted line). As is seen, OL behavior becomes more prominent with the increase of the tunneling effect; choosing a large value for the tunneling rate may lead to larger optical limiting efficiency (i.e., smaller optical limiting threshold). This point is further illustrated in Fig. 3b, which depicts the threshold transmission for the OL in the QDM. As is clear, the input intensity value corresponding to the onset of the limiting (i.e., optical power limiting threshold) is found to vary from 0.08 to 0.12 MW/cm$^{2}$. It is also apparent from this figure that the threshold value decreases with increasing the coherent tunneling so that it reaches about 0.8 $\times 10^{5}$ W/cm$^{2}$ for the case of $T_e$ = $\Gamma$; therefore, a prominent feature of an ideal optical limiter; i.e., low-limiting threshold, can be more readily extracted by choosing larger tunneling rates; it is interesting to note that the threshold of the limiting behavior would be a function of input voltage, allowing the optimization of the OL behavior. Notice that the limiting amplitude only showed slight variation with the increase of the tunneling rate, therefore, its corresponding plot is omitted.

**Figure 4:**
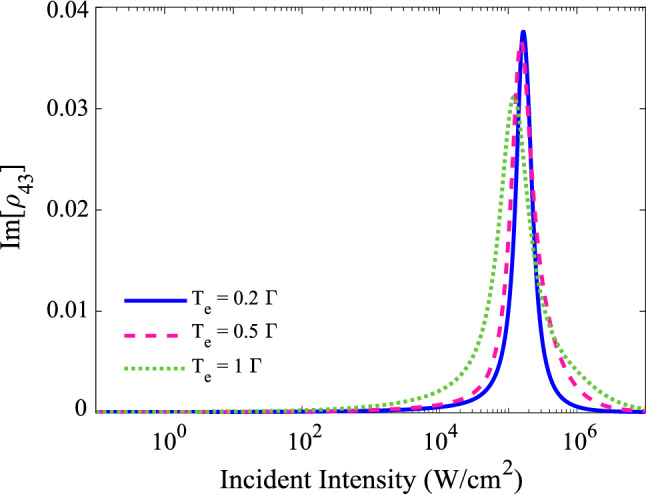
Tunneling-induced optical limiting is due to a reverse saturable absorption mechanism. The imaginary part of coherence $\rho _{43}$ versus the intensity of the incident field, for three different values of interdot tunneling: $T_{e}= 0.2\Gamma$ (solid line), $0.5\Gamma$ (dashed line) and $\Gamma$ (dotted line). The other parameters are the same as Fig. 2b.

Here, we are going to explain a physical interpretation of tunneling-induced OL on the basis of the RSA; Fig. 4 shows the imaginary part of coherence $\rho _{43}$ versus the input intensity for three different values of interdot tunneling: $T_{e}$= 0.2$\Gamma$ (solid line), 0.5$\Gamma$ (dashed line) and $\Gamma$ (dotted line). The other used parameters are the same as Fig. 2b. An investigation of this figure shows that a similar trend can be observed for all different tunneling rates: by increasing the input intensities into the OL region, the imaginary part of the probe transition coherence, and subsequently, the absorption of the probe field increase, leading to the generation of the RSA. To understand the physical origin of the RSA, we introduce the following analytical expression for the imaginary part of the probe transition coherence ($\mathfrak {Im}[\rho _{43}]$)-for the case of $\Gamma _{2}=\Gamma _{4}=\Gamma$, $\Delta _2=\Delta _4=\Delta$ and $T_{e}\simeq 1$-which can approximately follow the OL behavior:

5 $$\begin{aligned} \mathfrak {Im}[\rho _{43}]=\frac{8\,\Delta ^2 \,\Omega _c^2 \,\Gamma \, T_e^2 \,\Omega _p}{D}, \end{aligned}$$

where $D = 16 \Delta ^4 \,(T_e^{4} + \Delta ^4) + T_e^{4} \, \Omega _p^{2} \,(\Gamma ^2 + 4 \Delta ^2)-12 \Delta ^2 \, \Omega _p^{4}\,(\Delta ^2 +2 \Omega _c^2) + 4 \Omega _p^{6} \,(\Delta ^2 +3 \Omega _c^2)$. An investigation of Eq. (5) shows three important points as follows: (1) The interdot tunneling ($T_e$) has a major role in establishing the RSA mechanism. (2) The OL can not appear in the one-photon resonance condition. (3) The dominant nonlinear phenomenon for the OL behavior is the cross-Kerr nonlinearity which is applied via the transition channel, $\vert 3 \rangle \xrightarrow []{T_e} \vert 2 \rangle \xrightarrow []{\Omega _c^*} \vert 1 \rangle \xrightarrow []{\Omega _c} \vert 2 \rangle \xrightarrow []{T_{e}} \vert 3 \rangle \xrightarrow []{\Omega _p} \vert 4 \rangle$.

**Figure 5:**
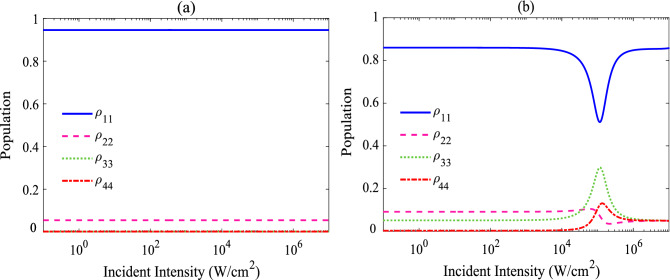
Effect of the tunneling on the population distribution of levels. Final steady-state population as a function of the intensity of the incident field, for the case of $T_{e}$= 0 in (**a**) and $T_{e}= \Gamma$ in (**b**). The other parameters are the same as Fig. 2b.

Now, we proceed to study the effect of the interdot tunneling on the population distribution of levels in order to gain a simple physical mechanism for such limiting behavior via the population transfer and their redistribution among the energy levels. Figure 5 shows the final steady-state population in different levels as a function of the intensity of the incident field, for the case of $T_{e}$ = 0 in (a) and $T_{e}$ = $\Gamma$ in (b). Noting that the used parameters are the same as Fig. 2b and it is assumed that the QDM is initially populated in ground state. As it is expected, in the absence of the interdot tunneling ($T_{e}$ = 0), the most of population is in the ground state; in fact, in the absence of the electron tunneling, the four-level QDM converts to two-independent two-level systems, and consequently, the population is accumulated in the ground state, because of the weak probe field. In the presence of the interdot tunneling and for the case of lower input intensities, the situation is somewhat similar to the previous case without tunneling effect; the state with the most population is the ground state. However, by increasing the input intensity into the OL region, the population transfer and the redistribution among the energy levels reveal a different trend: The population in the ground state may be drastically decreased and an accumulation of the population in two upper levels ($|3\rangle$ and $|4\rangle$) along with a slight depletion in the state $|2\rangle$, can be found. Moreover, by increasing the probe intensity, the population difference of state $|3\rangle$ and $|4\rangle$ grows and reaches a maximum value to induce the maximum absorption for the probe field in the OL region. Note that the small part of population traps in states $|2\rangle$ and $|3\rangle$, because of the interdot tunneling induced coherence, and does not have any contribution in the absorption of the probe field. Then, as it can be seen in Fig. 5b the population difference for weak probe region cannot induce any absorption and the system becomes transparent for the probe field.

**Figure 6:**
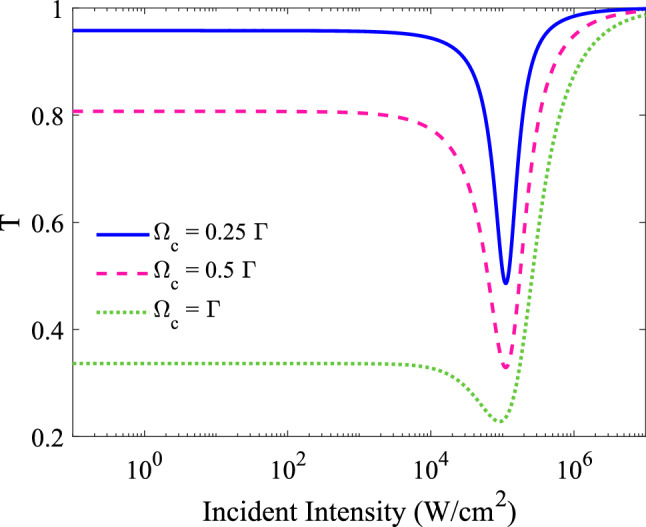
Effect of the Rabi frequency of coupling laser field on the optical limiting performance of the system. The variation of the transmittance as a function of the input intensity, for $T_{e}$ = $\Gamma$ in different Rabi frequency rates of coupling laser field, $\Omega _{c}=0.25 \Gamma$(solid line), $0.5 \Gamma$ (dashed line) and $\Gamma$ (dotted line). Other used parameters are the same as Fig. 3a.

The Rabi frequency of the coupling field is another useful parameter for controlling the OL properties of the system. The variation of the transmittance as a function of the input intensity are shown in in Fig. 6, for $T_{e}$= $\Gamma$ and different Rabi frequency rates of coupling laser field, $\Omega _{c}=0.25 \Gamma$ (solid line), $0.5 \Gamma$ (dashed line) and $\Gamma$ (dotted line) . Other used parameters are the same as Fig. 3a. By choosing various Rabi frequency rates of coupling laser field, the different behaviors can be seen for the optical limiting action. By increasing the Rabi frequency of coupling laser field, the limiting threshold and limiting amplitude decreases, but the linear transmittance simultaneously reduces to a small value, which doesn’t show appropriate behavior for optical limiting. Therefore, by examining the limiting amplitude and limiting threshold, the optimum OL can be obtained for $\Omega _{c}=0.5 \Gamma$.

**Figure 7:**
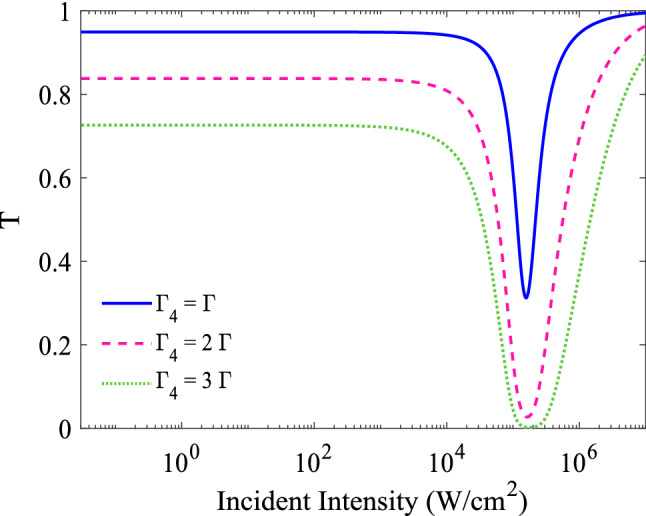
Effect of the decay rate on the optical limiting performance of the system. Transmittance versus the intensity of the incident field, for $T_{e}= 0.2\Gamma$ in three different decay rates of state $|4\rangle$: $\Gamma _{4}= \Gamma$ (solid line), $2\Gamma$ (dashed line) and $3\Gamma$ (dotted line). The other parameters are the same as Fig. 3a.

It seems that the decay rate of states can affect OL properties of the system. We are going to study the effect of the decay rate of the levels on the limiting threshold and limiting amplitude of the system. Figure 7 shows the variation in the normalized transmittance as a function of the input intensity for $T_{e}=\Gamma$ in three different decay rates of state $|4\rangle$: $\Gamma _{4}= \Gamma$ (solid line), $2\Gamma$ (dashed line) and $3\Gamma$ (dotted line). Other used parameters are same as in Fig. 3a. An investigation on Fig. 7 shows that increasing the decay rate of the state $|4\rangle$, has a constructive role in decreasing the limiting amplitude and limiting threshold, in comparison to the results of Fig. 3a.

**Figure 8:**
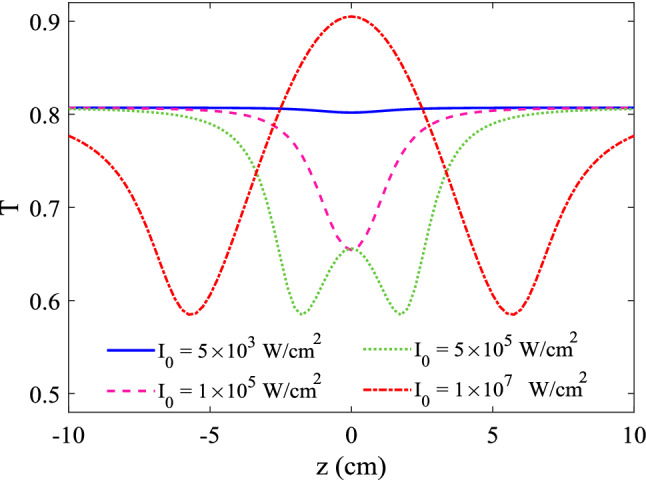
Z-scan results for various input intensities. Numerical results for the Z-scan transmittance as a function of the sample position (*z*), for different input intensities: $\hbox {5 kW/cm}^{2}$, $\hbox {0.1 MW/cm}^{2}$, $\hbox {0.5 MW/cm}^{2}$ and $\hbox {10 MW/cm}^{2}$. The other parameters are the same as Fig. 3a.

It is worth mentioning that, we ignore the size distribution and indeed consider a uniform QD molecule ensemble. Production of an ensemble of QDs with highest possible uniformity has a major role in generation of the limiting behavior. Several techniques have been introduced to reduce the inhomogeneous distribution of dot size^53^. Due to nonuniformity of QDs, laser fields experience various detunings during interacting with different QDs^54,55^. Then, the effect of size distribution on the OLs can be understood via Fig. 2a, in which the transmittance is plotted for different values of the detuning. The narrow size distribution can affect the optical limiting behavior, but it cannot destroy it because of the non-resonant necessary condition for establishing the limiting behavior.

In previous figures, the OL properties in the system are determined by plotting the transmittance versus the input intensity. Here, we proceed to discuss the features of the optical power limiting through a Z-scan technique with a laser field at 1.36 $\upmu$m. As noted above, in a Z-scan technique, a Gaussian laser beam propagating in the *z*-direction is focused to a narrow waist by using lens. The sample is moved along this direction and the transmittance of the tightly focused laser beam(s) is measured as a function of the *z* position; the sample experiences the maximum intensity at the focus, which gradually decreases in either direction from the focal point. An increase in the transmittance at the focus is assigned to the RSA mechanism, while the presence of SA action indicates a decrease in the transmittance near the focal point. Here, we have performed a simulation using a Gaussian laser pulse, propagating along +*z*, with an intensity of

6 $$\begin{aligned} I_{p}(z,r)=I_{0} \, \frac{w^{2}_{0}}{w^{2}(z)} \,\exp \left[ -\frac{2r^{2}}{w^{2}(z)}\right] , \end{aligned}$$

with $I_{0}$ being as the peak probe intensity. Also, $w(z)=w_{0}\,[1+(z/z_{0})^{2}]^{1/2}$ represents the beam radius at *z* (the distance of the sample from the focal point), $z_{0}=\pi w^{2}_{0}/\lambda$ and $w_{0}$ being as the diffraction length of the beam and the beam waist radius at the focus, respectively.

As noted previously, in the case of a typical RSA, the sample would experience the maximum absorption in the vicinity of the focal point (*z* = 0), giving rise to a dip in transmission near the focus; while, an SA action indicates a peak with respect to the focus in its accompanying transmission curve. Here, we present the numerical results for the Z-scan transmittance for different input intensities, with the same parameters of Fig. 3a. Figure 8 shows the Z-scan results for various input intensities: 5 kW/cm$^{2}$, 0.1 MW/cm$^{2}$, 0.5 MW/cm$^{2}$ and 10 MW/cm$^{2}$. At very low input intensity of 5 kW/cm$^2$, no variation is observed, however, the Z-scan curve for the intensity of 0.1 MW/cm$^{2}$ shows a clear valley, with a sudden decrease at the focus, which implies that a typical RSA action would be produced. The transmission curve for the case of $\hbox {0.5 MW/cm}^{2}$ shows two dips with a peak in the middle, implying that SA in the sample dominates nonlinear properties of the material. Also, the transmission of the sample at peak intensity of $\hbox {10 MW/cm}^{2}$ reaches about 0.9 at the focus, while it drops to 0.6 at dip positions.

In summary, to design the optimum OLs in visible-infrared band, the self-assembled (In,Ga)As/GaAs QDs with the radius of 9 nm and low intensity about $5\times 10^7\hbox { cm}^{-2}$ are used with optical confinement factor about $\Gamma _{\mathrm {opt}}=2.25\times 10^{-3}$ and the effective mode volume about $V=\hbox {11 nm}^{3}$. Noting that few nanometers inter-distance of QDs induces the maximum interdot tunneling. Also the frequency of applied laser fields should be tuned far from resonant excitation. Moreover, the intensity of the coupling field should be chosen about $10^{4}\hbox { W/cm}^{2}$ and the intensity region of optical limiting becomes less than $10^5\hbox { W/cm}^{2}$.

## Conclusions

In conclusion, a convenient way to generate the tunneling-induced optical power limiting behavior in a four-level QDM system has been proposed. Also, the effect of various parameters on the limiting effect has been investigated and it has been found that the system can reveal good OL properties in a certain intensity region. More interestingly, the threshold of the limiting behavior would be a function of the input voltage, allowing the optimization of the OL behavior; in fact, such a convenient control of the limiting action, along with the high linear transmittance at low intensities and the low-threshold OL behavior, may render these molecules promising for OL device development. Moreover, by investigating the absorption of the probe transition, it has been demonstrated that the tunneling-induced limiting can be attributed to the RSA mechanism; furthermore, analytical results show that the RSA is established via the cross-Kerr nonlinearity which is induced by the inerdot tunneling. Finally, a theoretical investigation of the Z-scan technique is presented to further characterization of the optical power limiting.
